# User Perceptions of an mHealth Medicine Dosing Tool for Community Health Workers

**DOI:** 10.2196/mhealth.2459

**Published:** 2013-04-04

**Authors:** Daniel Palazuelos, Assiatou B Diallo, Lindsay Palazuelos, Narath Carlile, Jonathan D Payne, Molly F Franke

**Affiliations:** ^1^Brigham and Women's HospitalDepartment of Medicine, Division of Global Health EquityBoston, MAUnited States; ^2^Harvard Medical SchoolDepartment of Global Health and Social MedicineBoston, MAUnited States; ^3^Partners in HealthBoston, MAUnited States; ^4^mHealth AllianceUnited Nations FoundationWashington, DCUnited States

**Keywords:** community health worker, cellular phone, mobile health, Mexico, Guatemala, decision support techniques, medical order entry systems

## Abstract

**Background:**

Mobile health (mHealth) technologies provide many potential benefits to the delivery of health care. Medical decision support tools have shown particular promise in improving quality of care and provider workflow. Frontline health workers such as Community Health Workers (CHWs) have been shown to be effective in extending the reach of care, yet only a few medicine dosing tools are available to them.

**Objective:**

We developed an mHealth medicine dosing tool tailored to the skill level of CHWs to assist in the delivery of care. The mHealth tool was created for CHWs with primary school education working in rural Mexico and Guatemala. Perceptions and impressions of this tool were collected and compared to an existing paper-based medicine dosing tool.

**Methods:**

Seventeen Partners In Health CHWs in rural Mexico and Guatemala completed a one-day training in the mHealth medicine dosing tool. Following the training, a prescription dosing test was administered, and CHWs were given the choice to use the mHealth or paper-based tool to answer 7 questions. Subsequently, demographic and qualitative data was collected using a questionnaire and an in-person interview conducted in Spanish, then translated into English. The qualitative questions captured data on 4 categories: comfort, acceptability, preference, and accuracy. Qualitative responses were analyzed for major themes and quantitative variables were analyzed using SAS.

**Results:**

82% of the 17 CHWs chose the mHealth tool for at least 1 of 7 questions compared to 53% (9/17) who chose to use the paper-based tool. 93% (13/14) rated the phone as being easy or very easy to use, and 56% (5/9) who used the paper-based tool rated it as easy or very easy. Dosing accuracy was generally higher among questions answered using the mHealth tool relative to questions answered using the paper-based tool. Analysis of major qualitative themes indicated that the mHealth tool was perceived as being quick, easy to use, and as having complete information. The mHealth tool was seen as an acceptable dosing tool to use and as a way for CHWs to gain credibility within the community.

**Conclusions:**

A tailored cell phone-based mHealth medicine dosing tool was found to be useful and acceptable by CHWs in rural Mexico and Guatemala. The streamlined workflow of the mHealth tool and benefits such as the speed and self-lighting were found to be particularly useful features. Well designed and positioned tools such as this may improve effective task shifting by reinforcing the tasks that different cadres of workers are asked to perform. Further studies can explore how to best implement this mHealth tool in real-world settings, including how to incorporate the best elements of the paper-based tool that were also found to be helpful.

## Introduction

Mobile health (mHealth) is a growing field that aims to utilize cell phone and tablet technologies to improve the delivery of health care. Pilot projects have demonstrated many potential benefits of mHealth tools, including improved patient attendance, adherence to antiretroviral medicines (ARVs), and provider adherence to treatment guidelines [1-5]. One type of mHealth tool in particular, the medical decision support tool, shows great promise in improving the quality of care provided to patients. Just as certain smartphone- or Internet-based technologies are increasingly being used by physicians to improve their work flow and quality of care [6], similar technologies may also be tailored to the skill levels of different cadres of frontline health workers (FLHWs) in a variety of settings to provide similar patient care benefits.

The community health worker (CHW) is one type of FLHW that is increasingly viewed as a viable provider of some basic primary care tasks in resource-poor settings. They have been recognized as potential agents of behavior change, as well as valuable health workers extending the reach of care [7]. Studies related to the Community Case Management (CCM) of childhood illnesses by CHWs have revealed that CHWs can assume significant clinical responsibilities traditionally reserved for nurses or physicians, including the diagnosis and treatment of common diseases [8-12]. Key factors in CHW success include adequate training and clear bedside support [13]. Although there are paper-based prescription and dosing resources occasionally available to FLHWs, such as the Nicaraguan book “Buscando Remedio” and the Hesperian Foundation book “Where There is No Doctor,” mHealth decision support tools have great potential to serve as critical references for CHWs, particularly for those working in remote areas. If well positioned and supported, CHWs may even form part of an alternative system to the unregulated medicine market that is common in many developing settings [14].

To facilitate medication prescription and dosing among community health workers in rural areas, the first author, while working with the Boston-based non-governmental organization Partners In Health (PIH), led the development of an algorithm-based medicine dosing reference mHealth software that can be run without cell phone connectivity on many commonly available Java-enabled mobile devices, including cell phones. This mHealth tool was created for use by CHWs who have only a primary school education, and is to our knowledge the first of its kind. Following an initial training session of the mHealth tool, we studied perceptions and impressions of this new technology, relative to an existing paper-based tool, among CHWs in two rural areas of Mexico and Guatemala.

## Methods

### Study Design

We designed the study to capture CHW perceptions of the mHealth dosing tool after an initial training aiming to introduce them to the new software. The study consisted of applying an original survey to, and conducting interviews with, participating CHWs in Mexico and Guatemala. The ethics committees of the Brigham and Women’s Hospital and Harvard Medical School exempted this study. Each CHW was individually consented to participate. The initial training lasted one full day, at no-cost to the CHWs, and was part of a larger CHW curriculum in rural primary care that included didactic lessons and bedside mentorship clinical sessions. Before the training, the CHWs were already familiar with the process of seeing patients and providing basic medicines for a limited number of well-defined clinical entities.

### Study Population and Setting

CHWs aged 16 or older from the PIH-supported projects in Mexico (N=11) and Guatemala (N=6) who participated in the initial mHealth tool training were eligible to participate in the study. All 17 participants agreed to enroll. CHWs were approved to use the mHealth tool in their daily work only upon demonstrating proficiency in the tool’s function and content; at the time of this writing, the tool was still not yet being used clinically.

The small mountain towns in which these CHWs operated were generally poor and isolated, with limited access to affordable primary health care. The Mexican towns were accessible only by dirt roads, which were often impassable for part of the year due to heavy rains. They had small under-resourced government clinics with private doctors in nearby cities providing basic urgent care on a fee-for-service basis. The Guatemalan towns were reachable by paved roads and public transportation and had small government clinics with private fee-for-service options in nearby cities. At the time of the study, there was no cell phone signal available in any of the Mexican towns, whereas there was a robust cell phone system throughout the region of Guatemala where the CHWs lived.

### Overview of the mHealth Tool

The cell phone-based mHealth tool utilized in this study was conceived and designed by DP and LP. Two volunteer programmers wrote the code for it to function on CommCare, an open source platform that was originally developed by the mHealth company, DiMagi Inc. The language of the program was targeted to a primary school reading level, and no calculations are required on the part of the user. The program runs on a “candy bar” Nokia phone to serve as a standalone, decision support mHealth tool. It does not require a cell network to function, but can be programmed to transmit data to, and interface with, electronic medical records or other such programs.

**Figure 1:**
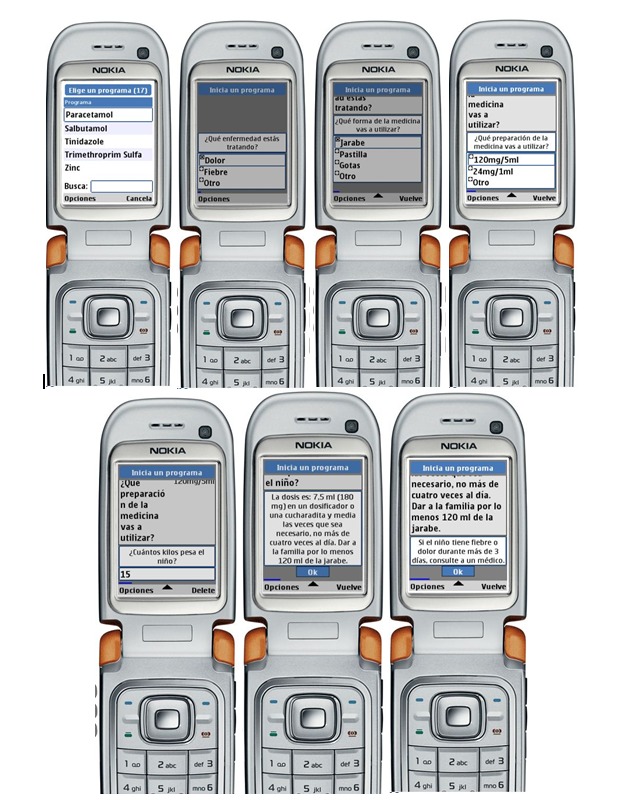
Screenshots of mHealth tool “chatter box” running through the Paracetamol algorithm for a child.

**Figure 2:**
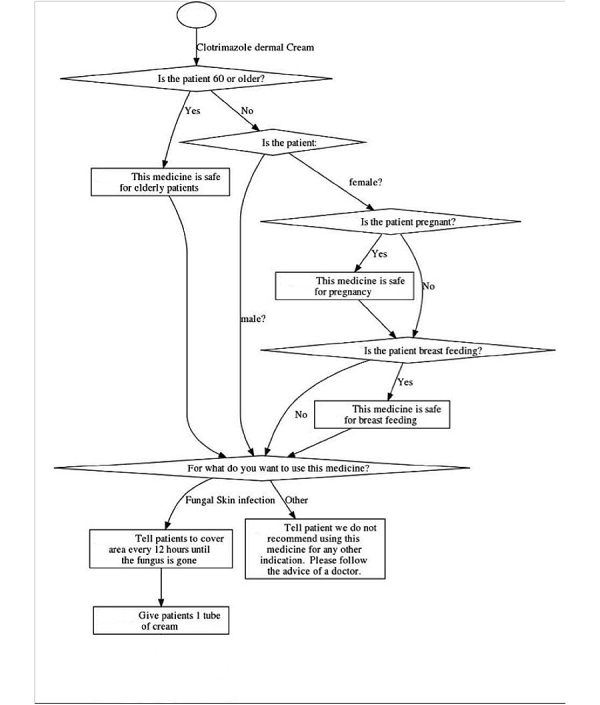
Sample medicine dosing algorithm (for a simple medicine–topical Clotrimazole cream).

The platform features easy programming and a distinctive “chatter box” feature (Figure 1), which allows the user to scroll through previous decisions without losing their place in the decision tree. The mHealth tool guides users through algorithm-based medication dosing (Figure 2), which accounts for patient characteristics including age, gender, pregnancy status, breastfeeding status, allergies, the ability to combine usage with other medicines, and weight (for pediatric dosing). Certain features of the program were unique due to the nature of the electronic format: the use of short-hand symbols (such as “<” for “less than”) was necessary due to space constraints, and greater specificity for the dosing categories was possible due to the ability to program as many advice points as desired (especially for weight-based dosing). Every algorithm follows a pre-determined clinical logic that limits the number of diseases for which a CHW can safely provide care. The clinical scenarios were taught in detail to the CHWs through their regular clinical training using easily understandable flow diagrams that could be referenced in a separate paper binder. Similarly, the medications included were limited to those that are essential and are adequately restocked through a typical supply chain. The program also specifies the quantity of medicine to give in order to assure a full course (eg, total number of pills, boxes, or bottles of syrup).

### Overview of the Paper-Based Tool

**Figure 3:**
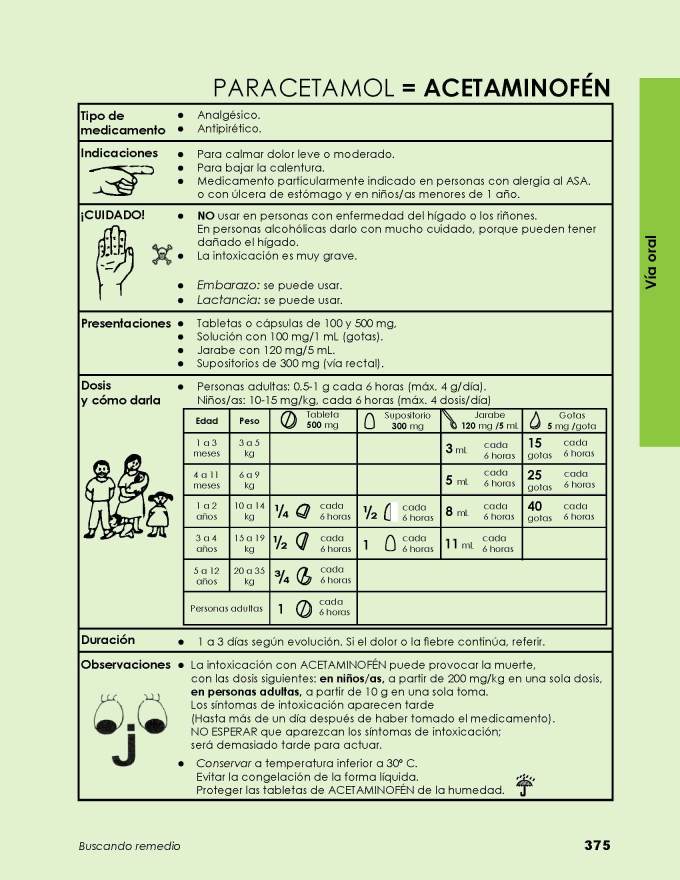
Single-page medicine fact sheet from “Buscando Remedio” (for Paracetamol).

The paper-based tool traditionally used by CHWs in the study settings is a book called Buscando Remedio (Spanish for “Searching for a Treatment”). It was written by the Nicaraguan government to serve as a primary care resource for FLHWs, and is freely available online [15]. The section primarily used in this study includes single-page information sheets for various medicines, including facts about the medicine, indications for use, cautionary advice, commonly available presentations, dosing instructions (often including a table that shows how much of the medicine to use in different weight categories), the duration of treatment, and a number of other miscellaneous comments (see Figure 3). These single-page sheets reference text presented in other sections of the book, with the expectation that users will access this information as needed.

### Data Collection

Each CHW completed an in-person interview that was administered in Spanish by DP and LP. MF and DP subsequently translated the interview notes into English. Data collection focused on 4 themes central to the adoption of a new technology in resource-poor settings: (1) comfort (ie, what were the participants’ assessments of the mHealth tool’s navigability and ease of use?), (2) acceptability (ie, what was the degree to which participants found the mHealth tool satisfactory and appropriate for use in dosing a medicine?), (3) preference (ie, did the participants tend to favor the mHealth tool over the existing paper-based tool when given a choice?), and (4) accuracy (ie, were the participants able to identify accurate dosing information utilizing the mHealth tool?).

The interview sought information on sociodemographic characteristics, previous experience as a CHW, and self-assessed experience with mobile technologies. To assess how accurately CHWs could produce dosing information using each tool, participants were asked to complete a practice test on dosing in which they could choose to use either the mHealth tool or the paper-based tool for each question. Following the practice test, we assessed their comfort with each tool by asking them to rate whether it was very easy, easy, hard, or very hard and to justify their tool selection choice. We assessed acceptability by asking the CHWs about their general impressions of the mHealth tool, and about specific aspects of the program that they did and did not like. Last, we asked CHWs to indicate which tool (the mHealth tool or the paper-based tool) would be their tool of choice when caring for 5 hypothetical patient populations (a child, a pregnant woman, an adolescent, an elderly woman, and an adult man from outside of the community) and to justify their decisions. DP and LP scored each question in the practice dosing test on a scale of either 1-4 or 1-6, depending on the content of the answer (2 points were given for the correctly stated amount of medicine that should be provided, 2 points for the correct schedule, and 2 points for the correct duration, if the medicine was more than a single dose and if the duration was explicitly stated in the text). Half credit, or 1 point, was given for partially correct elements. These scorings were performed independently by DP and LP, and then compared for accuracy. Few conflicts arose, but when they did they were discussed and resolved by agreeing on a common interpretation of how to apply the grading rules.

### Data Analysis

AD read the translated transcripts four times: first for familiarity, second for descriptive line-by-line coding, third for axial coding (isolating basic themes), and fourth for interpretive coding to capture the major specific concepts. From these codes, AD, MF, and DP agreed upon thematic variables and quantified the appearance of these variables in participants’ transcripts. They also observed overlap between the qualitative thematic and quantitative measured variables. The quantitative data was analyzed using SAS version 9.2.

## Results

### Participants

Characteristics of the 17 CHW participants are shown in Table 1. The majority of the CHWs (11/17, 65%) were recruited from Mexico, and the age distribution was roughly similar across the two countries. Although somewhat higher in Mexico than Guatemala, education level tended to be low overall, with no CHW having completed high school. Most participants (12/17, 70%) had 1 to 5 years of experience working as a CHW and this was similar across sites. While most CHWs had at least some experience using cell phones, one-third reported no prior experience.

### Comfort

The CHWs expressed comfort with the mHealth tool after the initial training session. 14/17 (82%) of CHWs elected to use the mHealth tool for at least 1 of the 7 questions on the practice test and 9/17 (53%) used the paper-based tool at least once. Of the 14 people who rated the phone application, 13 (93%) classified it as easy or very easy to use. Of the 9 people who rated the paper-based tool, 5 (56%) classified it as easy or very easy to use).

The remaining 4/9 CHWs (45%) who rated the paper-based tool as hard or very hard to use noted the lack of self-illumination with this tool and the length of time it took to find information (Table 2). A few participants noted that the paper-based tool allowed them greater flexibility to search the range of potential diagnoses and treatments.

[The book] has an index and it is complete.

[The book] is easy because the information is organized, alphabetized, and the graphics are easy to understand.

Narrative responses about the mHealth tool revealed that many CHW users enjoyed its ease, speed, and completeness.

[I liked it] because you enter the information and the result comes out. In the book you have to divide and multiply. The phone is much smoother.

Additionally, participants appreciated the mHealth tool because it afforded a light source for times when CHWs work in low light conditions (Tables 2 and 3).

Because of my age, I do not see well and with the phone I see better because it has light.

[The phone] will be better in an emergency because it is fast and has its own light (sometimes there is no electricity).

One potential drawback to the mHealth tool was highlighted by some CHWs, who were troubled by having to learn a new technology and information interface (Table 2).

### Acceptability

Overall, the CHWs in both countries accepted the mHealth tool as a satisfactory tool that was appropriate for use in dosing a medicine. Narrative responses about the mHealth tool again described this tool as being fast, easy, complete, and well organized (Table 3). In addition, some CHWs noted that using the mHealth tool on a phone would be a way to gain credibility in the community.

The people, upon seeing us look in the book, think badly of us. With the phone, they think we are important.

The phone is a more acceptable way to access information in front of the patient so as to not lose face.

Perceived challenges with the mHealth tool centered on the start-up investment needed to learn a new interface, and on the need to care for the technology that is more delicate and requires periodic maintenance (Table 3).

### Preference

**Figure 4:**
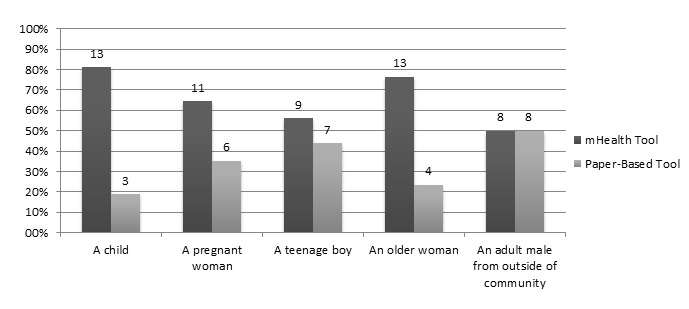
Tool preference, by potential patient demographic.

CHWs consistently demonstrated a preference for the mHealth tool over the paper-based tool in different clinical scenarios (Figure 4). Narrative responses about this preference revealed a number of themes surrounding how CHWs would capitalize on the factors unique to each tool to best interact with the patient receiving care (Table 4). For example, in providing care to someone not from the same community, one CHW would use the mHealth tool because it is “faster and easier. He comes from far away so he will probably want to return to his house right away.” On the other hand, in providing care to a pregnant woman, participants were divided between choosing the mHealth tool or the paper-based tool, depending on which they felt they were more likely to use correctly. For many, this was the first time that they had used the mHealth tool so they were concerned that without further practice and mastery of the tool, they were more likely to inadvertently misuse it and give a wrong dose. Others, who may have become more comfortable with using the new mHealth tool more quickly, would reach for it first when caring for a pregnant woman because they felt it more clearly stated “if a medication is okay or not”.

Participants who chose the paper-based tool in patient scenarios repeatedly cited the ample amount of information readily available as the reason for choosing this dosing tool. This may have been referring to both the book chapters available for further reading, and the ability to simultaneously view all related dosing information on the same page (which would be difficult with the mHealth tool as it provided information in a step-by-step fashion). They appreciated having more information available for their own learning and to better explain the condition and medication to their patient.

Because [the book] explains more, you can explain more and show [the patient] more information.

It is interesting to note that while some participants found the paper-based tool to provide more complete information than the mHealth tool, many also cited the completeness of the information in the mHealth tool. This was probably because both tools contained components that were perceived to provide all needed elements; the mHealth tool efficiently gave all pertinent clinical information by following a checklist, while the paper-based tool had more pictures and narratives about the diseases.

### Accuracy

**Figure 5:**
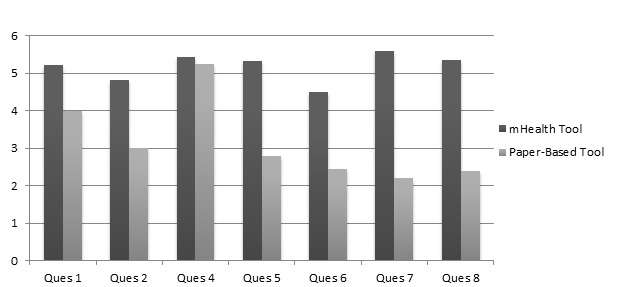
Mean dosing scores for practice test, by tool choice.

Use of the mHealth tool generally resulted in more accurate answers when compared to the paper-based tool. For 6 of 7 practice test questions, the mean score among those who answered with the mHealth tool was notably higher than the mean score among respondents who answered with the paper-based tool (Figure 5). In general, the difference was greatest in the questions that asked for pediatric doses based on age and weight, as opposed to standardized doses and courses for adults. Although not coded nor quantified, the majority of the errors with each tool followed a few general themes. For the paper-based tool, the CHWs often found it challenging to find the 3 different dosing elements needed (dose, schedule, and duration) as they were often in disparate locations without any clear pattern to follow. For the mHealth tool, the CHWs produced a wrong result if they inadvertently entered information incorrectly at some stage of the algorithm (ie, if they entered in a wrong gender, age, weight, etc).

**Table 1:** Characteristics of study population.

Characteristic (N^a^)		Total n (%)	Guatemala n (%)	Mexico n (%)
**Age**				
	18-25	6 (35)	3 (50)	3 (27)
	26-35	7 (41)	2 (33)	5 (45)
	36-45	3 (17)	1 (17)	2 (18)
	56-65	1 (6)	0 (0)	1 (9)
**Educational level (N=16)**				
	Some primary school	3 (19)	1 (20)	2 (18)
	Graduated primary school	5 (31)	3 (60)	2 (18)
	Some secondary school	3 (19)	0 (0)	3 (27)
	Graduated secondary school	3 (19)	0 (0)	3 (27)
	Some high school	2 (12)	1 (20)	1 (9)
	Graduated high school	0 (0)	0 (0)	0 (0)
**Years worked as community health worker**				
	<1	2 (12)	1 (16)	1 (9)
	1-5	12 (70)	4 (68)	8 (73)
	6-10	2 (12)	0 (0)	2 (18)
	>10	1 (6)	1 (16)	0 (0)
**Previous experience with cell phones**				
	None	6 (34)	2 (33)	4 (36)
	A little	4 (24)	2 (33)	2 (18)
	Some	4 (24)	2 (33)	2 (18)
	A lot	3 (18)	0 (0)	3 (27)

^a^Mexico N=11; Guatemala N=6, unless otherwise noted.

**Table 2:** Narrative responses about comfort/perceived ease of use of the 2 tools.

Response	mHealth tool	Paper-based tool
Very easy	*You enter the information and the result comes out. In the book, you have to divide and multiply. The phone is smoother.* *The doses are more complete, using milligrams instead of “a half” [of a tablet] as the book does.* *Because of my age, I do not see well and with the phone I see better because it has light.* *You press a button and then it gives you everything after just entering the name [of the medication].* *The questions all come packaged together and you do not have to look for them; it’s fast.* *You just have to look up the medication and it has for us the age and how many kilos and it tells us how much to give the patient.* *All of the information appears at once.*	*You just have to look up the medication and it tells you everything. It tells you the ages for the use of the medication and how much medication to give.*
Easy	*With just a name [of the medication] the phone designs and sees everything.* *You put in the information and everything appears.* *It is fast, like a calculator.* *Easy because it has all of the information.* *Enter the program [and immediately] look up the medication.* *You can look by age and it is easier (example: TMX_SMP for children). In the book, I imagine that is the same [but] it requires more attention (more concentration).*	*It has an index and is complete…and in order.* *It directs us to the pages to see.* *It is easy to look for information.* *It is easy because the information is organized, alphabetized, and the graphics are easy to understand.* *Everything is marked. Once you know how to use the book, it not necessary to learn another.*
Hard	*Searching via greater than ≥* or less than * ≤ [was difficult]…* the new concept * [was difficult to master immediately].* *I got a little confused with the information and it was a bit hard for me to manage [the phone].* *One does not know how to use it for the first time. It is very hard to learn it.*	*I got confused easily with the book—it does not explain well.* *I could not find information about pneumonia.* *We don’t know it well yet.*
Very hard	No responses	*It was dark; you cannot read the book well.* *The book is perfect but it takes longer—it’s also dark.*

**Table 3:** Narrative responses about acceptability of the mHealth tool.

Preference	Characteristic	Comment
**Like**		
	**Speed**	
		*Good, it is a big help because everything is faster.*
		*Yes, it is more practical, easier to use, and saves time.*
	**Perceived importance**	
		*Interesting; the people, upon seeing look in the book, think badly of us. With the phone, they think we are important.*
		*The phone is a more acceptable way to access information in front of the patient so as to not lose face.*
		*It is nice looking.*
	**Self-sufficient tool**	
		*It will be better in an emergency because it is fast and has its own light (sometimes there is no electricity).*
		*It has everything—it has how to transmit, you can take photos.*
	**Improves health provider confidence**	
		*It is good for us as health providers because we do not feel capable to give a consult, but with this book and the cell phone, we are going to be more confident that we can help the patient.*
	**Easy to understand**	
		*It is good because it has all of the information in a way that is easy to understand.*
		*I like it a lot because it is fast. If we can manage the phone, we can manage the book too.*
	**Complete Information**	
		*Yes, I like it because it is easier to use and gives more information.*
		*I liked all parts because everything had a place. I like it very much because it has all of the information for many examples (adults, children, age, weight, pregnant women, elderly).*
		*It tells you whether it [the medicine] can be used for pregnant women.*
	**Organization of tool**	
		*It is structured; it is very fast for looking things up. It seems very well written.*
	**Fun**	
		*It is more fun.*
**Dislike**		
	**Needs learning investment**	
		*It took time to learn it.*
		*I could not manage as it should be (as it would be by someone who knows). It is hard to learn to manage it.*
	**Technical difficulties**	
		*There is no signal here.*
		*It is blocked [asleep]; the letters are small.*
	**Requires more care**	
		*You have the commitment of taking care of it so that you don’t break it. You have to treat it delicately.*

**Table 4:** Narrative responses about tool preference in 5 hypothetical clinical scenarios.

Scenario	mHealth tool	Paper-based tool
A child	Self-sufficient tool *It saves time; you don’t need a pen or paper.* Multitask *While I am talking I can be looking at the dose, extracting information because it gives the information easily.* Well organized *The book is confusing (tangled)... The phone gives information fast and is easier to understand.* Exact dosing *It gives you the exact dose.* Provider confidence *I looked in the phone and then afterward in the book to see if it was correct, and it was!* *I can eliminate doubts quickly and later check in the book to see if they coincide.* More information *It has more information. Aside from the doses, it has more recommendations to make sure the parents understand.*	More information *It explains the disease and gives all of the information.* Time given to patient *Because it has more information about the illness (the phone may be so fast that you do not give time to the person and he/she may think badly about the appointment/consult.)* Easier to understand *I can understand [the book] better because the phone needs more [attention/comprehension/education].*
A pregnant woman	Speed and readability *Faster and easier to read.* Fear/confidence in tool *It can be used in pregnant women so as not to make a mistake because it tells me if a medication is okay or not.*	More information *More information about pregnancy.* Confidence in tool’s content/ fear in misuse *You can’t give a pregnant woman the wrong drug.* *If I use the phone badly, I could hurt a pregnant woman and her baby.*
A teenage boy	Easier to use *It is smarter, it tells you everything you need to know, it is more clear and well-explained.* *It is fast and easier to understand.* Fear/confidence in tool—verification *Depends on the disease. I will also check the book.* *The illness is probably mild so it can wait, I will verify it using both methods.* Ensure thoroughness *I like it because I am lazy to ask questions. It is hard for me to learn about the medications.*	More information *[The book] explains more so you can explain more and show [the patient] more information. The book has more information. But if they come in a hurry, I’m going to use the phone.* Fear/confidence in tool *Less risky to give him medications.”*
An old woman	Patient expectations *To know, it is more clear and well-explained. They come in a hurry and they say “If she starts reading, she must not know.”* *She is not going to be able to wait.* *It is a delicate case. You have to know to give her information. The phone has more information.* Well organized *The book is confusing (tangled) and confuses me. The phone gives information fast and is easier to understand.* Fear/confidence in tool—verification *Check in the phone and double check in the book.* *It tells you whether the medicine is good or bad if you are 60 years old.* *Because it talks specifically about the elderly*.	Patient demands/questions *I would prefer the book because she would want more information/explanation* More information *More information—it gives more information.*
An adult man not from your community	Fear/confidence *[The Phone] ensures that what I am thinking is correct at the time of giving the diagnosis.* Faster *Faster (he is coming from far away so will not want to wait).* *[The phone is] faster and easier; he comes from far away so he will probably want to return to his house right away.*	More information *Here I look for what type of illness he has, how old he is, and how long he has it and later depending on the illness and the pills, the book has indications and conflicting drugs.* Phone functionality *Sometimes the phones are not charged.* Learning *To continue learning more.* Use both *I am going to keep practicing a little more (the book), but it is always slower—I will probably use the two together but what happens if the phone breaks? Then I have the book and I understand it.*

## Discussion

### Key Findings

This study demonstrated that, following an initial introductory training session, the majority of CHWs were comfortable using a new cell phone-based mHealth tool, accepted the tool, and often preferred it relative to an existing paper-based dosing tool that they had been using. In a practice dosing test that asked CHWs to identify doses for certain conditions, accuracy tended to be higher among individuals who used the mHealth tool, relative to those who used the paper-based tool. With the growing interest in CCM and mHealth as resources available to improve and expand the performance of CHWs in resource-poor settings, this tool may be an important addition to the armamentarium for improving the quality and safety of care provided by such FLHWs.

The narrative responses collected about the 2 tools revealed a number of observations that will be important when implementing the mHealth tool in any large-scale program. For example, while many CHWs noted that the mHealth tool quickly provided the information needed, they enjoyed the expansive amount of information provided in the paper-based tool as it allowed them to continue learning on their own and gave them more information to share with patients. Knowing this, future users of the mHealth tool may also be concurrently provided with a variety of patient education sheets that will deepen and broaden the amount of information available to the CHW. It is also possible that the mHealth tool and these education sheets could be integrated so that there are bidirectional links between them.

One factor that most likely contributed to the usability of the mHealth tool was its streamlined workflow, designed to achieve effective task shifting by guiding CHWs to accurately dose medications for only a limited list of primary care ailments. The paper-based tool, on the other hand, was written to be understandable by a variety of providers but is not tailored for use by one cadre in particular; since it includes information on a wider range of medications and clinical entities, it will likely present information to some groups that is beyond their scope of practice. Simplified but well designed tools, such as this mHealth tool, may clarify and reinforce the tasks reserved for different FLHW cadres, be it CHWs, nurses, or doctors, so that each can achieve mastery in their sphere.

Indeed, an important feature of the mHealth tool’s platform is the ability to transparently customize the medicines, indications, and dosages in the mobile dosing software based on the local context. CHWs in different regions work with different formularies of medicines in their kit, and it is important that the system can be easily adapted to local needs so that it remains streamlined for that health worker’s workflow. The mobile dosing software has been designed to use a simple plain text programming language that can be quickly tailored locally as needed. In the future, it could also be modified and linked to medication inventory, potentially saving busy FLWHs from laborious paper reports.

Even though the use of the mHealth tool was observed to improve dosing accuracy, errors were still made with both tools. The goal for the clinical use of any tool should be close to 100% accuracy, so future iterative improvements will have to be made in order to achieve this goal. This can be done through a variety of strategies. First, while the mHealth tool already has a “chatter box” function that allows the user to review the data previously entered, further safety confirmation screens can be programmed in order to assure that one small slip does not lead to erroneous advice in the end. Next, implementers can provide simulated clinical encounters for CHW trainees in order to assure that they master the tool’s interface before using it with patients.

### Limitations

This study was conducted among a small group of CHWs working at 2 project sites where the mHealth tool was to be implemented as part of routine care. The small number of participants precludes us from drawing formal statistical comparisons between the mHealth and paper-based tools, and between the participants at the two sites. Because the mHealth tool was introduced as part of programmatic activities, the individuals who led the software development also conducted the trainings and research interviews. While it is possible that this may have resulted in a social desirability response bias if participants felt pressured to speak positively about the mHealth tool, we minimized this to the greatest extent possible by setting a tone of trust during interviews, encouraging participants to speak freely without fear of judgment, and assuring that their opinions would not affect their standing in the program. Lastly, participants were able to select which tool they wanted to use for each practice test question. If participants who better understood dosing in general tended to select the mHealth tool, this could at least partly explain higher accuracy scores for questions answered by this tool; however, this seems unlikely given that nearly all participants used the mHealth tool at least once during the practice test.

### Conclusions

The mHealth tool described in this study is one of many similar new technologies that are poised to make important contributions to how care is delivered in a variety of contexts. While the observations reported suggest that tools such as these hold great promise, further studies will be needed to assure that use of the mobile tools in actual clinical contexts improves outcomes. These studies should not try to answer a binary question about whether or not the tool is useful, but instead should explore how the tool can be improved and personalized to function best in its unique context. Similarly, appropriate technology supports, such as hand-crank chargers, solar chargers, and protective cases to prevent equipment damage, will also need to be utilized in order to maximize on-site functioning.

## Abbreviations

ARV: antiretroviral medicines
CCM: Community Case Management
CHW: community health worker
FLHW: frontline health workers
PIH: Partners In Health
